# Biological Evaluation of Thermosensitive Hydrogels of Chitosan/Hydrolyzed Collagen/β-GP in an In Vitro Model of Induced Cardiac Ischemia

**DOI:** 10.3390/polym16152206

**Published:** 2024-08-02

**Authors:** Lina Orozco Marín, Yuliet Montoya, John Bustamante

**Affiliations:** 1 Tissue Engineering and Cardiovascular Prosthetics Line, Cardiovascular Dynamics Group, Bioengineering Center, Universidad Pontificia Bolivariana, Medellín 050004, Colombia; lina.orozcom@upb.edu.co (L.O.M.); john.bustamante@upb.edu.co (J.B.); 2 Working Committee of Cardiovascular Bioengineering, Colombian Society of Cardiology and Cardiovascular Surgery, Bogotá 1013, Colombia

**Keywords:** thermosensitive hydrogels, biomaterials, tissue regeneration, cardiac ischemia

## Abstract

Ischemic events can culminate in acute myocardial infarction, which is generated by irreversible cardiac lesions that cannot be restored due to the limited regenerative capacity of the heart. Cardiac cell therapy aims to replace injured or necrotic cells with healthy and functional cells. Tissue engineering and cardiovascular regenerative medicine propose therapeutic alternatives using biomaterials that mimic the native extracellular environment and improve cellular and tissue functionality. This investigation evaluates the effect of thermosensitive hydrogels, and murine fetal ventricular cardiomyocytes encapsulated in thermosensitive hydrogels, on the contractile function of cardiomyocyte regeneration during an ischemic event. Chitosan and hydrolyzed collagen thermosensitive hydrogels were developed, and they were physically and chemically characterized. Likewise, their biocompatibility was evaluated through cytotoxicity assays by MTT, LDH, and their hemolytic capacity. The hydrogels, and cells inside the hydrogels, were used as an intervention for primary cardiomyocytes under hypoxic conditions to determine the restoration of the contractile capacity by measuring intracellular calcium levels and the expressions of binding proteins, such as a-actinin and connexin 43. These results evidence the potential of natural thermosensitive hydrogels to restore the bioelectrical functionality of ischemic cardiomyocytes.

## 1. Introduction

Ischemic events in cardiac tissue can lead to cell damage or death due to oxygen and nutrient deprivation in an injured area, and extracellular matrix micro-architecture organization is also affected when the cells are affected [1]. However, unlike most tissues in the human body, in which a tissue regeneration process is initiated due to tissue-resident stem cells, in cardiac tissue, the population density of stem cells is lower, and they have a potentially limited differentiation. For this reason, the regenerative capacity of the heart cannot regenerate the number of cardiac cells lost after an ischemic event [2].

Within the alternative therapies used for the traditional treatment of cardiac ischemia, new therapies based on thermosensitive hydrogels have attracted the attention of the scientific community because they represent a minimally invasive therapy alternative [3].

Hydrogels, being 3D structures, can retain large amounts of water, increasing their biocompatibility and biodegradability, which is essential for tissue regeneration [4]. Thermosensitive hydrogels represent a therapeutic strategy that can be administered through catheterization in an in vivo model, thanks to the fact that the polymeric compounds that compose it can respond to changes in temperature by transforming from solid to gel states [5]. For cardiac therapy applications, this sol-gel transition occurs at a body temperature of 37 °C to initiate its gelation process at the target site [5].

Due to the complexity of the heart, since it is a muscle in a constant contraction state, an essential characteristic of biomaterials is that they must promote electrical conductivity; they must also be suitable for integrating cardiomyocytes or other cell types into their structure to compensate for the loss of cells and mechanotransduction signals due to cardiac injury [6,7].

Based on the abovementioned issues, this research aims to develop a natural thermosensitive hydrogel and assess its physical, chemical, and biological characterizations. The hydrogel is subsequently used in biological assays as a scaffold for the cell encapsulation of primary cardiomyocytes to restore cell contractile activity in cells under induced ischemic conditions, to be considered as an alternative therapeutic strategy for cardiac tissue regeneration.

## 2. Methods

### 2.1. Natural Biocomposite Hydrogel Development

Solutions of low-molecular-weight chitosan (Ch 50-190 KDa, degree of deacetylation of 75–85%) at 2.0 and 2.5% *w*/*v* in 0.1 N of acetic acid (HAc) were prepared under magnetic stirring at 340 rpm for 24 h. A solution of β-glycerolphosphate (β-GP) was prepared with an initial concentration of 50% *w*/*v* in cold H_2_Odd, and solutions of hydrolyzed bovine collagen type B (HcB) and porcine type A (HcP) were prepared at a concentration of 2% *w*/*v* in 0.02 N of Hac, both under magnetic stirring conditions at 200 rpm for 2 h at room temperature (RT).

The first step was performed to determine the volumetric ratio of Ch/β-GP. For this stage, Ch solutions at different concentrations of 2.0 and 2.5% *w*/*v* were mixed with β-GP at final concentrations of 6.5, 7.5, and 8.5% *w*/*v* under magnetic stirring at 4 °C for 30 min. In a liquid state, the hydrogels were stored at 4 °C for 12 h for degassing. Gelation was performed by incubating the samples at 37 °C in a thermal bath to determine their gelation times using the inverted tube method and the turbidity of the solution [8,9].

The second step was to determine the volumetric ratio of Hc, leaving the Ch/β-GP ratios as described in the previous step fixed. The Ch/β-GP solution was mixed with HcB or HcP at concentrations of 1.5, 2.5, 3.5, 4.5, and 6.5% *w*/*v*. The solution was stirred at 4 °C for 15 min. Finally, β-GP was added to the solution and mixed for 30 min. Once the hydrogels were obtained in a liquid state, they were degassed, and the gelation time was determined using the inverted tube method and the turbidity of the solution. The ionic conductivity of the resulting solutions was determined using a potable conductivity meter (Apera instruments EC400S) (Columbus, OH, USA).

### 2.2. Hydrogels’ Physicochemical Characterization

#### 2.2.1. Scanning Electron Microscopy (SEM)

The morphological characteristics of the hydrogels were determined using samples in a gelled state. The samples were subjected to successive ethanol baths, starting at an initial concentration of 50% *v*/*v* and ending at a final concentration of 100% *v*/*v*. Subsequently, they were taken to the desiccator to remove any excess moisture, and then they were cryo-fractured with N_2_. The samples were analyzed with a scanning electron microscope (Jeol NeoScope JCM-6000plus, Peabody, MA, USA) operated at 15 kV, with a 1000× magnification.

#### 2.2.2. Fourier Transform Infrared Spectroscopy with an Attenuated Total Reflectance Modulus (ATR)

To determine the changes in the functional groups, present in the developed biomaterial, the hydrogels in the gelled state were taken to the desiccator to remove any moisture. The samples were characterized using an FTIR-ATR spectrophotometer (Nicolet iS50, Waltham, MA, USA) with an attenuated total reflectance module) at a resolution of 4 cm^−1^ and 32 scans were performed.

#### 2.2.3. Mass Loss Assay

The behavior of the hydrogel in contact with the physiological medium was evaluated using samples in a gelled state, which were submerged in Hank’s solution at pH 7.4 for 17 days at 37 °C. Records of the hydrogel mass were obtained at the following times: 0 h (*W*_0_), 1 h, 2 h, 3 h, 4 h, 5 h, 1 day, 2 days, 3 days, 7 days, 10 days, 14 days, and 17 days (*W_t_*). The mass loss of the hydrogels is calculated using Equation (1), where *W_t_* is the mass at any time, *t*, and *W*_0_ is the initial mass of the sample:

(1) $$H_{w}=W_{t}-W_{0}$$

### 2.3. Hydrogel Biocompatibility

#### 2.3.1. In Vitro Model: RL-14 Human Fetal Ventricular Cardiomyocytes

Biocompatibility assays were performed with the fetal human ventricular cardiomyocyte cell line (RL-14) obtained from the ATCC (PTA-1499). This cell line was obtained from a postmitotic primary culture transformed with the SV-40 monoclonal sequence. The cells were cultured with DMEM, penicillin/streptomycin, and 10% FBS under controlled conditions of CO_2_ (5%), O_2_ (95%), relative humidity (95%), and temperature (37 °C). The culture medium was changed every 48 h, and a new subculture was performed when the cells reached a confluence higher than or equal to 85%.

#### 2.3.2. Hydrogel Sterilization

Each liquid hydrogel was exposed to ultraviolet radiation in a type-II biological safety laminar flow cabinet (ESCO Sc2-4a2, Singapore) for 30 min for sterilization and subsequent use in biological assays.

#### 2.3.3. Hydrogel Cytotoxicity

The cytotoxicity degree of the hydrogel was determined through cell cultures with the RL-14 cardiomyocyte cell line in contact with the experimental samples. For this, 50 µL/well of each type of hydrogel was placed in a 96-well plate and incubated at 37 °C. Once the hydrogels had gelled, they were washed with 0.9% NaCl and unsupplemented DMEM. Subsequently, 3 × 10^3^ cells/well of RL-14 were seeded in 80 µL of DMEM. The cytotoxic effects of each hydrogel were evaluated at 24, 48, and 72 h post-intervention, using the MTT assay according to ISO 10993-5 [10]. This method consisted of the metabolic reduction of [3-(4,5-dimethylthiazol-2-yl)-2,5-diphenyltetrazole] (MTT) bromide by the mitochondrial enzyme succinate dehydrogenase, converting it into a purple compound called formazan. The number of living cells was proportional to the optical density (OD) derived from the formazan produced [10]. The cytotoxicity of the hydrogel is presented in terms of the cell viability expressed as OD using Equation (2):

(2) $$Cell\;viability\;\left(OD\right)=\frac{\left(ODP-ODCM\right)\;}{\left(ODCC-ODM\right)}\times 100$$

where *ODP*: cells + precursor optical density, *ODCM*: precursor + medium optical density, *ODCC*: optical density cell control, and *ODM*: optical density control medium. Additionally, there was a positive control (C+) for cell death (cells treated with 15% *v*/*v* peroxide).

#### 2.3.4. Cell Viability

The subculture of the supernatants from the cells in contact with the surface of the hydrogel was a second method used to determine cell viability. For this, a 50 µL/well of each type of hydrogel was placed in a 96-well plate and incubated at 37 °C. Once the hydrogels were gelled, they were washed with 0.9% NaCl and unsupplemented DMEM. Subsequently, 3 × 10^3^ cells/well of RL-14 were seeded in 80 µL of DMEM with 10% FSB and incubated at 37 °C with 5% CO_2_. After 8 days, the supernatant from each well was collected and subcultured into new wells. The same procedure was performed on the 16th day.

A total of 24 h after the subculture of each supernatant, the medium was changed to remove the cells that did not adhere to the culture dish. Micrographs of the cell growth were obtained from the subcultured supernatants starting on day 2, and every 4 days until the well was at least 80% confluent using an inverted-light microscope (IM3FL4).

#### 2.3.5. Hydrogel Hemolytic Capacity

The hemolytic capacity of the developed hydrogels was evaluated according to ISO10993-4 [11]. Bovine blood was obtained from the Medellín City, Colombia, slaughterhouse and collected in BD Vacutainer^®^ tubes (Franklin Lakes, NJ, USA) with EDTA. The Health Research Ethics Committee of the Universidad Pontificia Bolivariana approved the use of bovine blood (approval date: 30 September 2019; certification number: 127).

Before the assay, the hydrogels were incubated in NaCl at 37 °C for 30 min, and the blood was diluted in NaCl in a 4:5 ratio. Subsequently, the hydrogels were immersed in the diluted blood and incubated at 37 °C for 1 h. After this time, the hydrogels were removed and the blood from each sample was centrifuged at 2500 rpm for 5 min. Finally, the plasma was carefully separated and analyzed in a Lambda Bio 10 spectrophotometer at a wavelength (*λ*) of 545 nm. The controls used were *PC*: positive control for hemolysis (blood in H_2_Od 4:5) and NC: negative control for hemolysis (blood in NaCl 4:5). The percentage of hemolysis was calculated using Equation (3):

(3) $$\%\;Hemolysis=\frac{\lambda hydrogel-\lambda NC}{\lambda PC-\lambda NC}\times 100$$

### 2.4. Biological Evaluation of the Hydrogel in an In Vitro Model of Induced Ischemia

#### 2.4.1. Isolation and Culture of Primary Cardiomyocytes

The in vitro model was based on cultures of primary cardiac cells isolated from mice with the BALB/c strain, using the modification of the methodology reported by Vidyasekar et al. [12]. To isolate the cardiac cells, neonatal mice 1–3 days old were sacrificed by decapitation, and the hearts were extracted by a sternotomy, and washed in cold HBSS. The hearts were cut into small pieces, incubated with trypsin, and inactivated with an isolation culture medium. A total of 200 µL of collagenase was added for 30 min, and subsequently, the solution was resuspended with medium to break up the tissue. Cell viability was determined by trypan blue assay. Finally, the cells were used in two ways: encapsulated in the hydrogels or, for each functional assay, seeded in fibronectin-treated culture dishes. The culture medium was changed 24 and 48 h after seeding. Primary cardiac cells were cultured under controlled conditions of 37 °C, 5% CO_2_, 95% O_2_, and 95% relative humidity until 60–70% confluence.

#### 2.4.2. Cell Encapsulation

Cell encapsulation was performed, isolating and culturing primary cardiomyocytes to a 60–80% confluence. Subsequently, the cells were detached from the culture plate using trypsin at a concentration of 0.075% and incubated for 3 min at 37 °C. Finally, 7 × 10^3^ cells were mixed with 50 µL of hydrogel in a liquid state. This mixture was evenly arranged in 24-well culture plates and used as an intervention method in different experimental designs.

#### 2.4.3. Ischemia Model: Glucose Decrease and Hypoxia Induction

A hypoxia model was adapted from Hafez et al. [13] to emulate the physiological conditions of an ischemic event. Initially, neonatal cardiomyocytes were exposed to low glucose concentrations, changing the maintenance culture medium to glucose, serum, and phenol red-free medium (ischemic medium). Subsequently, the cell cultures were exposed for 15 min to low concentrations of O_2_ (1%) and CO_2_ (5%) and high concentrations of N_2_ (94%) in a hypoxic chamber (stem cell Technologies, 27310). Under these same conditions, the cells were incubated at 37 °C for 3 h.

After glucose deprivation and hypoxia induction, the cells were divided into 3 groups: the first group was incubated in normoxic conditions of 37 °C, 5% CO_2_, 95% O_2_, 95% relative humidity, and a maintenance medium; the second group was intervened with the hydrogels; and the third group was intervened with primary cells encapsulated in the hydrogels.

#### 2.4.4. Lactate Dehydrogenase (LDH) Cytotoxicity

LDH is an enzyme usually present in the cytoplasm of living cells and is released into the cell culture medium by the membrane permeabilization of severely affected or dead cells. The increased LDH activity in the culture supernatant is proportional to the number of lysed cells [14].

LDH levels were determined using the commercial kit MAK066 (Sigma-Aldrich, München, Germany). For the assay, the supernatant of the cell cultures was collected and centrifuged at 600 RCF for 10 min. Then, 10 µL of the supernatant was transferred to a 96-well plate with 40 µL/well of the LDH reaction solution. The mixture was incubated at 37 °C at 70 rpm for 30 min in the dark. The absorbance was measured in a spectrophotometer at a λ of 450 nm. The absorbance of the culture medium was used as a negative control and the medium of cells lysed with the kit’s lysis solution as a positive control.

LDH measurements were performed on healthy cells, cells under hypoxic conditions, and cells under hypoxic conditions intervened either with hydrogels, encapsulated cells, or under normoxic conditions. The cytotoxicity percentage was calculated using Equation (4):

(4) $$\%\;Cytotoxicity=\frac{\lambda \;sample-\lambda \;negative\;control}{\lambda \;positive\;control-\lambda \;negative\;control}\times 100$$

#### 2.4.5. Evaluation of the Cell Structural Changes

Fluorescence microscopy was used to determine the expressions of specific binding proteins, such as α-actinin and connexin 43 (Cx43). For this, primary cells were fixed with 4% formaldehyde for 15 min at room temperature, permeabilized with Triton X-100 (St. Louis, MO, USA), and blocked with 1% bovine serum albumin for 1 h. Subsequently, the cells were incubated with the primary antibodies α-sarcomeric actinin and Cx43, washed with PBS 1X, and incubated with the secondary antibody Alexa Fluor 488 for 1 h. The nuclei were stained with Hoechst. Finally, the samples were analyzed using a microscope with an OPTIKA brand fluorescence module, model IM-3FL4.

The structural changes were studied in healthy cells, cells under the hypoxic conditions, and cells under hypoxic conditions intervened either with hydrogels, encapsulated cells, or under normoxic conditions.

#### 2.4.6. Intracellular Calcium Measurements

An optical mapping system using fluorescent probes was implemented to measure the calcium currents in primary cardiomyocyte cell cultures. Indo-1 AM dye was used for the calcium measurements, a radiometric probe that was excited at λ 346 nm and emitted at λ 475 nm.

The cell cultures were labeled with calcium probes according to the protocols indicated by the distributors. Finally, the fluorescence intensity was measured for cultures with confluences greater than 75% and with cell densities of 2.5 × 10^4^, which were recorded with an inverted microscope with an OPTIKA brand fluorescence module, model IM-3FL4, and a recording camera brand OPTIKA PRO-5 (Ponteranica, Italy).

The intracellular calcium measurements were performed on healthy cells, cells under the process of ischemia, and cells under the process of ischemia treated with hydrogels, encapsulated cells, or under normoxic conditions.

### 2.5. Statistical Analysis

The result analysis was performed through an experiment design using a multifactorial ANOVA and different test statistics in the Statgraphics 18^®^ Centurion program, which allowed for establishing the best results in terms of cell viability, hydrogel hemolytic capacity, cell functionality, and calcium flux. The trials were performed with an experimental n of 3. Statistical differences were considered at a *p*-value < 0.05.

## 3. Results and Discussion

### 3.1. Natural Biocomposite Hydrogel Development: Determination of the Volumetric Ratios of Chitosan, β-Glycerolphosphate, and Hydrolyzed Collagen

Ch was evaluated at an initial concentration of 2% *w*/*v*, mixed with β-GP at an initial concentration of 50% *w*/*v* and final concentrations of 6.5, 7.5, and 8.5% *w*/*v*.

**Figure 1:**
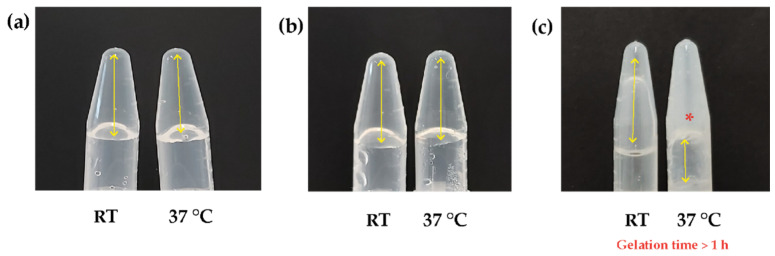
Determination of the gelation times of Ch/β-GP hydrogels at different β-GP concentrations. (**a**) β-GP 6.5% *w*/*v*, (**b**) β-GP 7.5% *w*/*v*, (**c**) β-GP 8.5% *w*/*v*. Double-headed yellow arrow: empty space. Asterisk: gelation. RT: room temperature.

The gelation time results are shown in Figure 1. The samples with β-GP at 6.5% *w*/*v* (a) and 7.5% *w*/*v* (b) descended down the tube both at RT and 37 °C and did not turn into a turbid solution, indicating the polymer mixture was preserved in a liquid state after 5 h without transitioning to the gelled state. Therefore, the samples were discarded as they did not present gelation.

In this type of material, the turbidity indicates the sol-gel transition. This behavior occurs because, in Ch, the distribution of deacetylated zones in the polysaccharide chains is not present along the entire carbon chain (random) but is distributed in blocks due to the type of chitin deacetylation [15]. In the gel formation process, an interaction occurs between the deacetylated zones, which are positively charged by the Ch acid solution. These deacetylated zones form hydrophilic domains following the interaction with negatively charged β-GP. On the other hand, the acetylated zones interact, forming large hydrophobic domains, even though there is a degree of deacetylation greater than 80%. In these hydrophobic domains, the light scatters due to their block distribution and size, which is finally observed as turbidity in the solution [9].

The sample containing β-GP 8.5% *w*/*v* (c) did not descend in the tube at 37 °C and exhibited turbidity, indicating gelation at a time longer than 1 h. However, this study aimed to develop a system that could provide structural support in vivo, retain encapsulated cells, and increase viability in ischemic heart tissue; 1 h was a long gelation time that could avoid the desired application [16,17].

**Figure 2:**
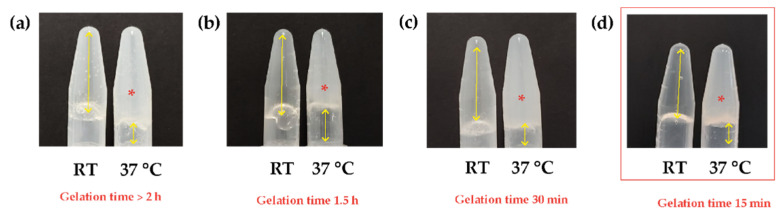
Determination of the gelation times of Ch/β-GP hydrogels at different β-GP concentrations. (**a**) β-GP 6.5% *w*/*v*, (**b**) β-GP 7.5% *w*/*v*, (**c**) β-GP 8.5% *w*/*v*, (**d**) β-GP 10.5% *w*/*v*. Double-headed yellow arrows: empty space. Asterisk: gelation. Orange square: selected sample. RT: room temperature.

According to the previous results, the Ch initial concentration was modified, increasing to 2.5% *w*/*v*, and mixed with the same β-GP concentrations under the same experimental conditions. In Figure 2, it can be observed the samples gelled with all the β-GP concentrations. As the β-GP increased, the gelation time decreased, so an additional concentration of β-GP at 10.5% *w*/*v* was included to achieve a shorter gelation time. The sample with β-GP 8.5% *w*/*v* was completely gelled after 30 min at 37 °C, while the sample with β-GP 10.5% *w*/*v* gelled in 15 min.

**Figure 3:**
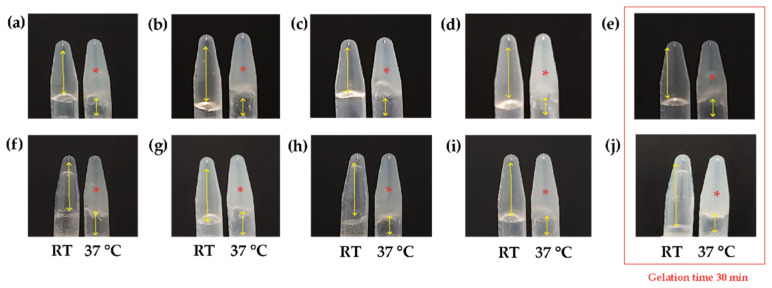
Determination of the gelation times of Ch/β-GP/Hc hydrogels at different Hc concentrations. (**a**) HcB 1.5% *w*/*v*, (**b**) HcB 2.5% *w*/*v*, (**c**) HcB 3.5% *w*/*v*, (**d**) HcB 4.5% *w*/*v* (**e**) HcB 6.5% *w*/*v* (**f**) HcP 1.5% *w*/*v*, (**g**) HcP 2.5% *w*/*v*, (**h**) HcP 3.5% *w*/*v*, (**i**) HcP 4.5% *w*/*v*, (**j**) HcP 6.5% *w*/*v*. Double-headed yellow arrows: empty space. Asterisk: gelation. Orange square: selected samples. RT: room temperature.

Once the Ch/β-GP ratio was determined, only the hydrogels with the shortest gelation times (β-GP 10.5% *w*/*v*) were mixed with HcB or HcP, and their gelation times were determined based on the concentration of the type of Hc. The results are shown in Figure 3, where it can be observed that the gelation time varies depending on the concentrations of HcB or HcP.

**Figure 4:**
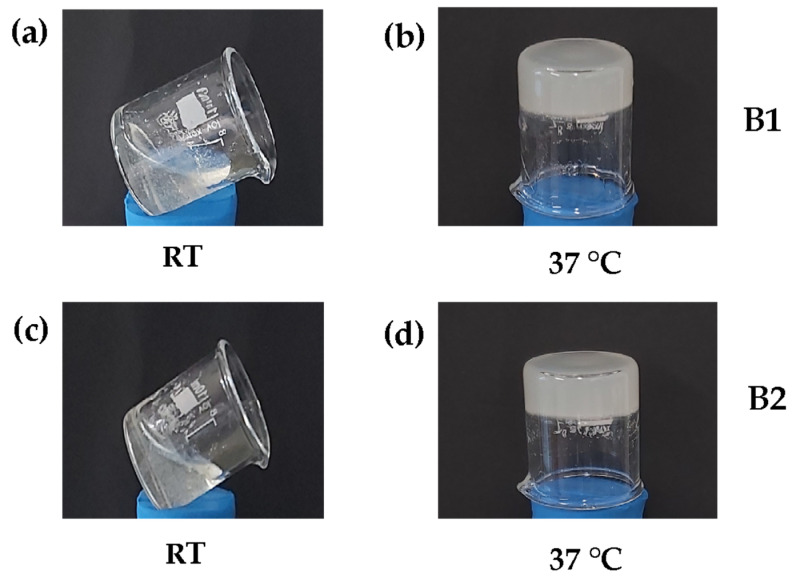
Thermosensitive Ch/β-GP/CH hydrogels. (**a**) Hydrogel B1 at room temperature (RT), (**b**) hydrogel B1 at 37 °C, (**c**) hydrogel B2 at room temperature (RT), (**d**) hydrogel B2 at 37 °C. B1: Ch 2.5% *w*/*v*/β-GP 10.5% *w*/*v*/HcB 6.5% *w*/*v*. B2: Ch 2.5% *w*/*v*/β-GP 10.5% *w*/*v*/HcP 6.5% *w*/*v*.

As a result, the hydrogels that previously presented a gelation time of 15 min (β-GP 10.5% *w*/*v*) increased to 30 min with the addition of HcB or HcP. Considering that this type of biomaterial was viewed as a therapeutic strategy, we pretended to create hydrogels with gelation times under than 60 min and with as many collagen fibers as possible to favor cellular interrelations. Therefore, hydrogels with HcB and HcP at a concentration of 6.5% *w*/*v* were chosen. In conclusion, two types of hydrogels, named B1 and B2, with different concentrations of β-GP and different Hc natures were obtained, as shown in Figure 4.

The hydrogels showed ionic conductivity results of 19.8 ± 0.4 mS/cm and 20.4 ± 1.8 mS/cm. Considering the cell typology of the application, which was cardiomyocytes that exhibited a contractile capacity, an ionic imbalance permitted the polarity of the plasma membrane to change and trigger mechanical coupling. The conductivity observed allowed us to clarify that the hydrogels could interact with the physiological syncytium providing them with the potential to travel [18].

### 3.2. Physicochemical Characterization of Hydrogels

#### 3.2.1. Scanning Electron Microscopy (SEM)

**Figure 5:**
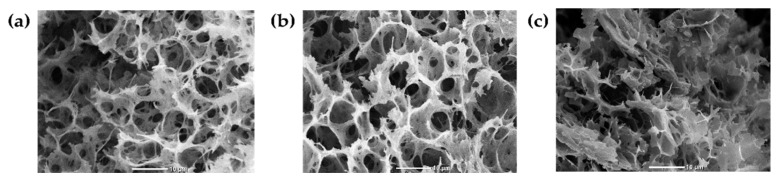
Representative micrographs of hydrogel micro-architecture. (**a**) B1: Ch 2.5% *w*/*v*/β-GP 10.5% *w*/*v*/HcB 6.5% *w*/*v*, (**b**) B2: Ch 2.5% *w*/*v*/β-GP 10.5% *w*/*v*/HcP 6.5% *w*/*v*, (**c**) Ch 2.5% *w*/*v*/β-GP 10.5% *w*/*v*. Scale bar: 10 µm.

Figure 5 presents hydrogel electronic micrographs, where B1 (a) and B2 (b) present a three-dimensional internal micro-architecture with interconnected pores.

In tissue engineering applications, it is essential to generate structures similar to the micro-architecture and composition of the ECM of native tissues. The ECM is a polymeric network that allows cells to communicate through their cytosolic membranes with the surrounding environment [3].

An interconnected porous structure favors cell encapsulation by supporting cell anchoring and retaining processes. In addition, it facilitates nutrient and oxygen exchange and the metabolite release, allowing for the preservation of a suitable micro-environment for cell–cell division, differentiation, and interaction [19].

According to the qualitative analysis, the HcB (a) samples presented a better-defined porous framework than those with HcP (b). This may be because HcB has shorter polypeptide chains (40–90 kDa) due to the chemical treatment use to obtain it, favoring the generation of a tight and compact network, while HcP has longer polypeptide chains (100–700 KDa), which may create a framework with labile, ample, and loose pores [20].

Likewise, the presence of Hc influences the hydrogel’s micro-architecture, as shown in the micrograph (c), where a hydrogel without Hc can be observed. The structure has fewer defined pores than the samples with Hc due to the reduced electrostatic attraction between the polymer chains limiting crosslinking. In conclusion, both the presence and nature of Hc affect the hydrogel’s internal micro-architecture.

#### 3.2.2. Fourier Transform Infrared Spectroscopy with Attenuated Total Reflectance Modulus (ATR)

**Figure 6:**
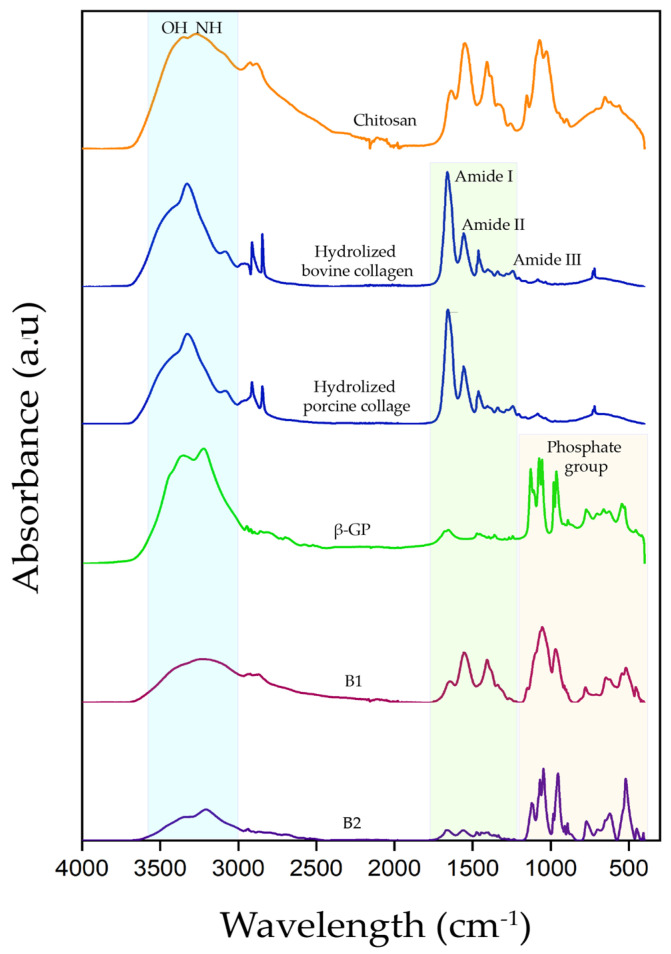
FTIR-ATR absorption spectra of hydrogels and their precursors. B1: Ch 2.5% *w*/*v*/β-GP 10.5% *w*/*v*/HcB 6.5% *w*/*v*. B2: Ch 2.5% *w*/*v*/β-GP 10.5% *w*/*v*/HcP 6.5% *w*/*w*.

Figure 6 shows the absorption spectra for B1 and B2 hydrogels and their precursors. The absorption spectra of the two types of hydrogels exhibited changes in the intensity and shape of the bands that corresponded to the O–H and N–H groups concerning the precursors, which was related to the formation of hydrogen bonds [8].

At the same time, the amide I (C=O), II (N–H and C–N) and III (N–H and C–N) bands were conserved at wavelengths similar to those observed for the HcB and HcP controls. However, these bands decreased in intensity in the gelation process, indicating that the amides participated in the gelation reaction, probably forming intramolecular or intermolecular hydrogen bonds with β-GP, Ch, and water, or electrostatics attractions between amino and carboxyl groups [8]. The interaction was also evident by the slight displacements of β-GP bands with respect to the hydrogel samples, corresponding to the bonds P=O (from 1130 cm^−1^ to 1128 cm^−1^), O–P–O (from 1057 cm^−1^ to 1053 cm^−1^), P–O–H (from 1057 cm^−1^ to 105 cm^−1^), P–O (from 983 cm^−1^ to 982 cm^−1^), and O–P–O (from 544 cm^−1^ to 524 cm^−1^).

These results show the interaction between the three system compounds, allowing us to obtain a thermosensitive hydrogel from a natural origin.

#### 3.2.3. Mass Loss Assay

**Figure 7:**
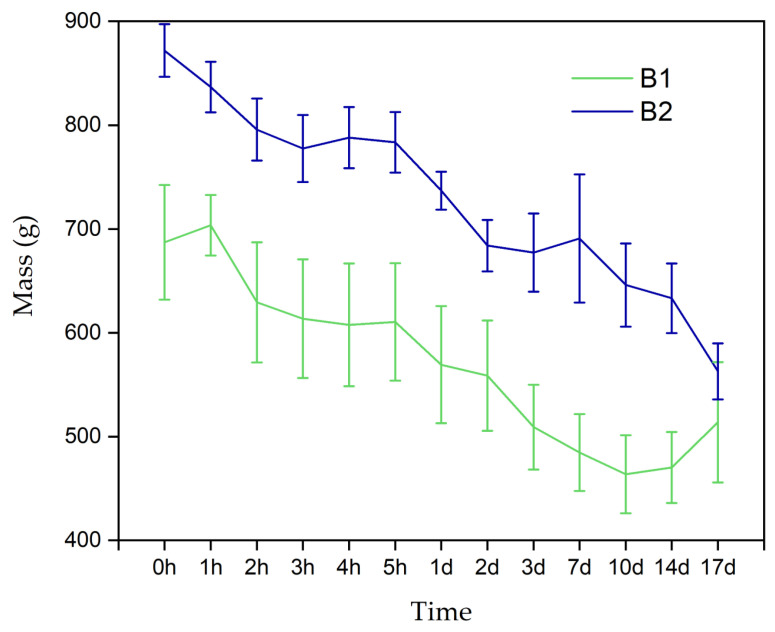
Mass loss evaluation over 17 consecutive days for hydrated hydrogels. B1: Ch 2.5% *w*/*v*/β-GP 10.5% *w*/*v*/HcB 6.5% *w*/*v*. B2: Ch 2.5% *w*/*v*/β-GP 10.5% *w*/*v*/HcP 6.5% *w*/*v*.

Figure 7 shows the behavior of the hydrated hydrogels B1 and B2 in contact with a physiological medium for 17 days. The graph shows how the samples lost mass, with slight increases over time. This behavior may be directly related to the medium’s entry into the hydrogel’s porous structure, and can represent the flow of nutrients and oxygen, metabolite exit, and cellular release [8].

### 3.3. Hydrogel Biocompatibility

#### 3.3.1. Hydrogel Cytotoxicity

**Figure 8:**
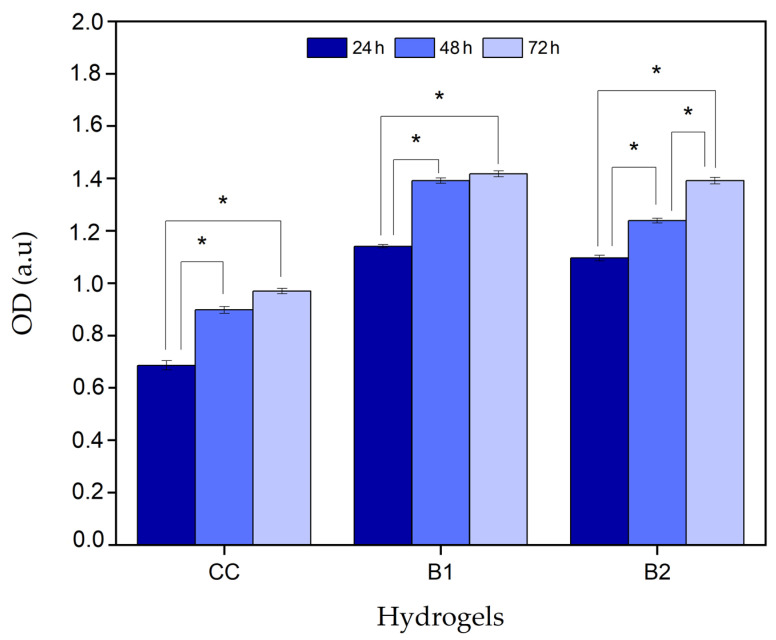
Cytotoxic effects of hydrogels in an in vitro model of RL-14 cardiomyocytes. CC: cellular control. B1: Ch 2.5% *w*/*v*/β-GP 10.5% *w*/*v*/HcB 6.5% *w*/*v*. B2: Ch 2.5% *w*/*v*/β-GP 10.5% *w*/*v*/HcP 6.5% *w*/*v*. * *p*-value < 0.05.

The MTT assay determined the cytotoxicity values of the B1 and B2 hydrogels. Cell viability was represented as OD, as shown in Figure 8. The OD is directly related to the absorbance of the sample, which, at the same time, indicates the formazan compound concentration in the medium.

Samples B1 and B2 presented a statistically significant increase in OD levels between 24 and 72 h. B1 increased from 1.14 at 24 h to 1.42 at 72 h, and B2 from 1.1 at 24 h to 1.40 at 72 h. This result indicates that the cells in contact with the hydrogels for 72 h do not lose their viability; conversely, they can proliferate over time and are metabolically active to transform the MTT compound.

The cells in contact with both hydrogels exceeded the cell viability of the control cells. This effect occurred because β-GP enhanced the percentage of cell viability by 130% compared to the control cells (100%), with statistically significant differences. Ahmadi and Bruijn also reported this behavior, where bone marrow-derived mesenchymal cells increased their proliferation by 20% when in contact with Ch 2% *w*/*v* and β-GP 10% *w*/*v* [21]. On the other hand, β-GP was also used in cell cultures as a source of inorganic phosphate (Pi) for the osteogenic differentiation of bone marrow stromal cells [22]. In in vivo models, Pi is an essential nutrient for organisms since it is required as a component of energy metabolism in the signaling pathway of kinases and phosphatases, which are involved in the entire cell division cycle [23]. Pi is also involved in the synthesis and function of lipids, carbohydrates, and nucleic acids [24]. Therefore, in this investigation, β-GP could have been a source of Pi and participated in intracellular reactions necessary for cell proliferation.

The B1 hydrogel exhibited greater cell viability over time compared to B2. The differential component between both hydrogels Hc, where its polypeptide chains varied depending on the acid or basic extraction method [20]. HcP has longer chains and a high molecular weight, between 100 and 700 Kda, generating a higher-viscosity solution, whereas HcB has shorter chains and an average molecular weight, between 40 and 90 Kda, generating a lower-viscosity solution than HcP [20]. In this case, the B2 hydrogel had HcP in its composition, generating greater viscosity in the cell culture medium, directly affecting the availability of the nutrients in the medium and mass transfer, limiting cell proliferation and metabolic responses [25] which was the reason why B2 hydrogel cell viability was lower compared to B1.

On the other hand, the average molecular mass of Hc is proportional to the Bloom index, which measures the force necessary to create a depression in a specific area of the gel [20]. It has been shown in vitro that the higher the Bloom index, the lower the cytocompatibility and the greater the inflammatory reaction [26]. Theoretically, HcP presents a high index of 300, which is related to the more significant effect of reducing cell viability, and HcB presents a minor reduction in viability with a medium index of 225 (Figure 8) [20].

#### 3.3.2. Cell Viability

The supernatant subculture was created to observe the proliferation of RL-14 human fetal ventricular cardiomyocytes, which were in contact with the B1 and B2 hydrogels for 16 days. The evaluation time was determined, considering the differences between 2D and 3D cultures, including the slowing down of cell division due to the non-homogeneous oxygen and nutrient availability throughout the cell culture [27].

**Figure 9:**
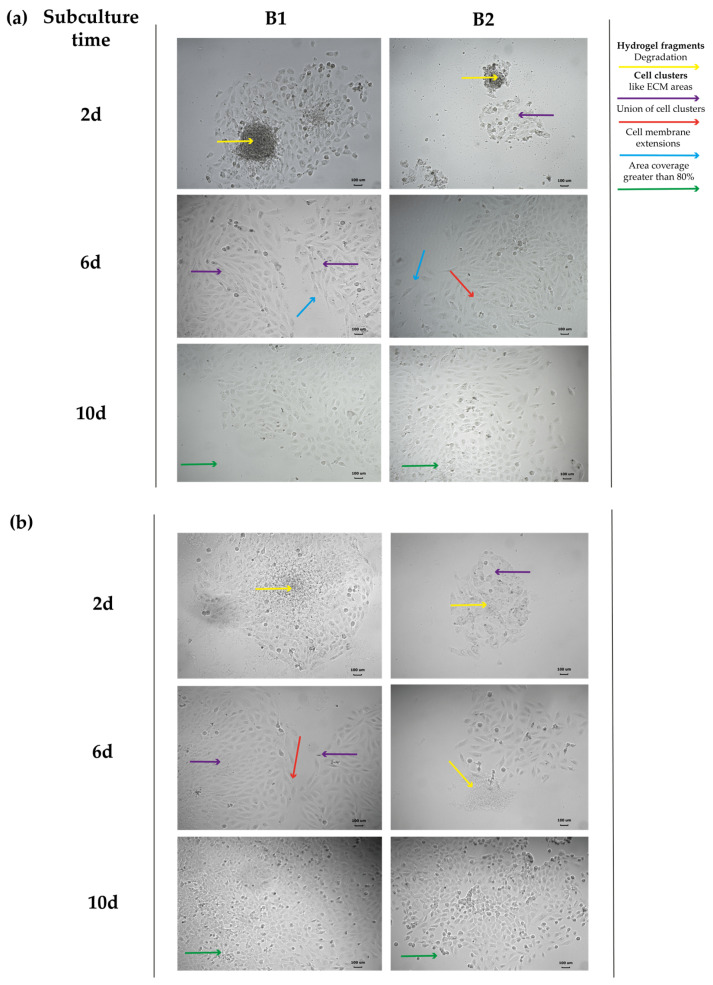
Proliferation of RL-14 human fetal ventricular cardiomyocytes from supernatants of cells interacting with thermosensitive hydrogels for (**a**) 8 and (**b**) 16 days; 10× magnification. B1: Ch 2.5% *w*/*v*/β-GP 10.5% *w*/*v*/HcB 6.5% *w*/*v*. B2: Ch 2.5% *w*/*v*/β-GP 10.5% *w*/*v*/HcP 6.5% *w*/*v*. Scale bar: 100 µm.

Figure 9 presents the cell culture micrographs for each type of hydrogel. These supernatants presented cells and hydrogel fragments that increased over time, indicating their progressive degradation in a biologically active micro-environment with living cells performing metabolic processes. The biomaterial degradation is a positive aspect, considering that the healthy encapsulated cells in its structure can be released to facilitate the regeneration of myocardial injury [28]. These hydrogel fragments in the cell subculture act as an ECM, favoring cardiomyocyte union and stimulating their proliferation until they join to each other and cover the entire cell culture surface. In addition, at the morphological level, long extensions of the cell membrane are observed, such as lamellipodia and filopodia, which are not usually seen in a 2D culture. The presence of Ch and Hc in the hydrogel fragments probably stimulated native ECM synthesis and, therefore, a cell–ECM interaction since Ch components, such as N-acetylglucosamine and D-glucosamine, were reported to induce type-I collagen synthesis [29]. Likewise, they generated diverse phenotypes like those of a native cellular micro-environment [30,31].

In some investigations, biocompatibility tests have been performed over short periods (24–78 h). Therefore, the observed effect is limited to the first hours after the exposure of the cells to the hydrogel. In contrast, in this investigation, the hydrogels’ biocompatibility was observed for up to 16 days, when the cells still retained the ability to proliferate and be functional.

#### 3.3.3. Hydrogel Hemolytic Capacity

The ISO 10993-4 standard established the hemocompatible tests, which allowed for the quantitative measurement of free hemoglobin in blood plasma [11]. Increased plasma hemoglobin correlates with red blood cell lysis and reflects the fragility of the erythrocyte membrane in contact with thermosensitive hydrogels [32]. Table 1 indicates the hemolytic index and degree for biomedical devices or alternative therapeutic strategies [33].

**Figure 10:**
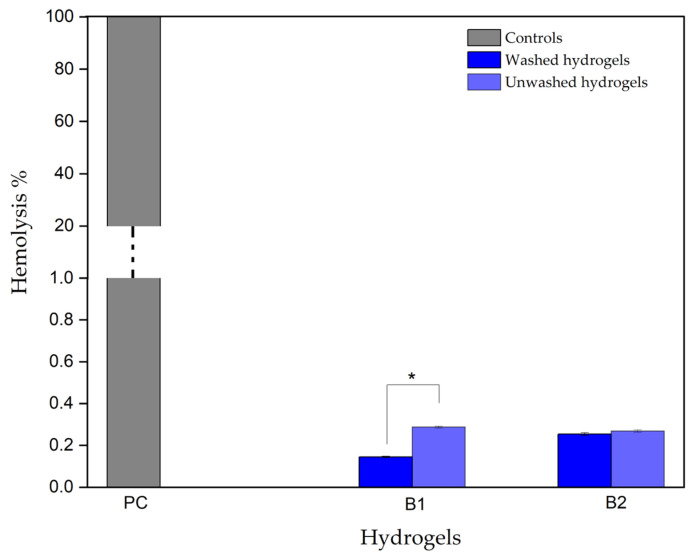
Hydrogel hemolytic capacity. PC: Positive control. B1: Ch 2.5% *w*/*v*/β-GP 10.5% *w*/*v*/HcB 6.5% *w*/*v*. B2: Ch 2.5% *w*/*v*/β-GP 10.5% *w*/*v*/HcP 6.5% *w*/*v*. * *p*-value < 0.05.

Figure 10 shows the hemolytic capacity values of hydrogels B1 and B2. Two samples were evaluated, some previously washed with a saline solution and others without washing, since it has been reported that, after gelation, hydrogels can contain traces of β-GP that does not bind to polymer chains and can decrease the cell viability [8].

The graph shows that samples B1 and B2, which were washed before the test, have hemolysis percentages between 0.14% and 0.25%, respectively, and the samples that were not washed have hemolysis percentage between 0.28% and 0.27%, respectively. According to Table 1, both types of samples are classified within the established range as “non-hemolytic” and do not exhibit statistically significant differences (*p*-value < 0.05). This means it is optional to wash hydrogels B1 and B2 after gelation, which is one of the difficulties encountered when using this type of biomaterial. Since hydrogel implantation assumes a liquid state, it is necessary to prevent the hydrogel washing step since the polymeric interactions can be altered, thus affecting the gelation time and mechanism.

In conclusion, hydrogels B1 and B2 did not exhibit hemolytic behavior, a process related to red blood cells lysis, which can directly affect the capacity of the circulatory system to transport oxygen to body tissues, increasing arterial and pulmonary pressure levels and toxicity due to the release of the heme group, in addition to stimulating adverse effects, such as platelet, hemostatic, and immune system activation [34].

### 3.4. Biological Evaluation of Hydrogels in an In Vitro Model of Induced Ischemia

#### 3.4.1. Hypoxia Model and Cell Characteristics by Ischemia 

Understanding ischemic heart disease is critical to understanding the biological mechanisms that improve therapeutic approaches for restoring cardiomyocyte function after an ischemic event [13]. For this reason, different experimental models have been developed using neonatal cardiomyocytes, because, unlike adult cardiomyocytes, these cells present greater viability in culture, capable of forming a monolayer, thus allowing the study of cell junctions and bioelectrical conductivity [35].

One of the consequences of the progressive reduction in ATP in response to decreased oxygen and substrate deprivation in the ischemic process is damage to the composition, structure, and function of cell membranes, including the mitochondrial membrane [36]. In vitro studies have shown that this membrane damage can decrease contractile activity since calcium influx is affected, reactive oxygen species are increased, and cell death can occur by apoptosis and necrosis [37].

#### 3.4.2. Lactate Dehydrogenase (LDH) Cytotoxicity

To experimentally determine the conditions of hypoxia and glucose deprivation like those in an ischemic event, the lactate dehydrogenase (LDH) enzyme concentration was determined. This enzyme is essential for energy production and is found in almost all body tissues, including the heart. When cell damage occurs, LDH is released into the extracellular medium and enzyme levels increase, indicating that the cells have suffered irreversible damage to their cytoplasmic membranes [38].

**Figure 11:**
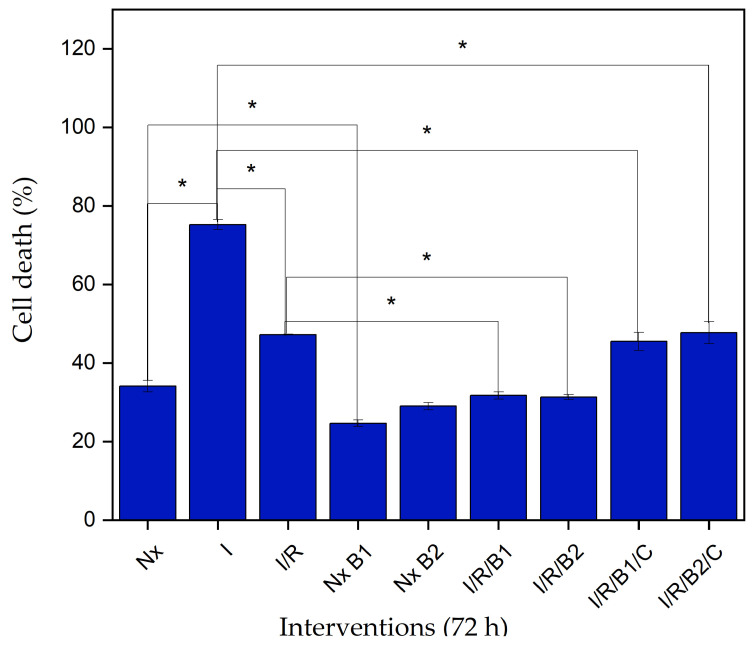
Cytotoxicity percentages determined by the LDH of primary cardiomyocytes under conditions of normoxia (Nx), ischemia (I), ischemia/normoxia (I/R), normoxia with hydrogels B1 or B2 (NxB1 or NxB2), ischemia/intervention with B1 hydrogel (I/R/B1), ischemia/intervention with B2 hydrogel (I/R/B2), ischemia/intervention with encapsulated cells in B1 hydrogel (I/R/B1/C), ischemia/intervention with encapsulated cells in B2 hydrogel (I/R/B2/C). * *p*-value < 0.05.

Figure 11 shows the cell death percentage after a hypoxia period and hydrogel intervention. Under normoxia conditions at 72 h, the cells showed a 34% cell death outcome. This behavior is associated with the normal dynamics of the culture, where, in the growth phase, some cells die and others slowly divide [39], resulting in basal LDH levels.

Following the ischemic event, the cell death percentage increased to 75% because the peroxidation membrane and caspase activation increased the cell membrane permeability and induction of inflammation. This process affects the cation influx regulation and causes cell swelling, leading to membrane rupture and the consequent release of LDH [39].

Ischemic cultures were subjected to normoxia conditions for 72 h or intervened with in two ways: B1 and B2 hydrogels in sol states or encapsulated the cells in hydrogels. Under ischemia/reperfusion (I/R) conditions, the cultures recovered by 28% compared to the ischemia control. However, when compared with normoxic cultures, a 13% increase in the percentage of cell death was observed, that is, the reperfusion process did not reach the minimum concentrations of LDH seen in healthy control cells. On the other hand, it was found that the ischemic cell cultures that were intervened with using hydrogels exhibited cell death percentages of 31.7% for B1 and 31.3% for B2, both lower than the healthy control cells, that is, both types of hydrogels had a positive effect on the cell viability after the ischemic event. This behavior can be related to the presence of β-GP in the hydrogels, representing a source of Pi, which participated in intracellular reactions necessary for cell division [22].

The same effect was seen for the cells that were not exposed to the ischemia process but treated simultaneously with B1 (NxB1) and B2 (NxB2) hydrogels. These cells showed a decrease in cell death rates compared to the healthy control cells, indicating that the hydrogels alone could reduce cell death rates under normoxic conditions.

In cultures intervened with encapsulated cells, a decrease in the cell death percentage was evident with respect to the I/R control. B1 presented a 42.4% cell death value in these conditions and B2 42.5%. These percentages were not lower than the healthy control cells; however, increasing the cell number (culture cells + encapsulated cells) was expected to increase the amount of LDH released into the medium. The cell death difference between the hydrogels and encapsulated cells in the hydrogels was approximately 11%; however, this difference was not statistically significant.

#### 3.4.3. Evaluation of Cell Structural Changes

##### Connexin 43

The cell membrane presents cell junctions that allow cell–cell and cell-extracellular matrix communications. This communication allows cell syncytia to maintain mechanical and bioelectrical integrity by exchanging physicochemical information [40].

There are different types of intercellular junctions, many of which are formed by transmembrane proteins. Gap junctions are essential to cardiac muscles since the bioelectrical signal that induces contractions moves rapidly between the cells through these junctions as ions, allowing the cells to contract independently and synchronously [41].

Gap junctions are formed by bringing connexons together. Each connexon, or hemichannel, comprises six protein subunits called connexins (Cxs). Cx43 is expressed predominantly in ventricular cardiomyocytes, followed by Cx45 [42]. Cx43 is expressed in the sarcolemma, nucleus, and mitochondria of cardiomyocytes. Mitochondria are abundant in cardiac cells representing 30% of their volume [43]. In mice, 78.4% of cardiomyocytes are multinucleated cells, and in humans, 25.5% [44].

**Figure 12:**
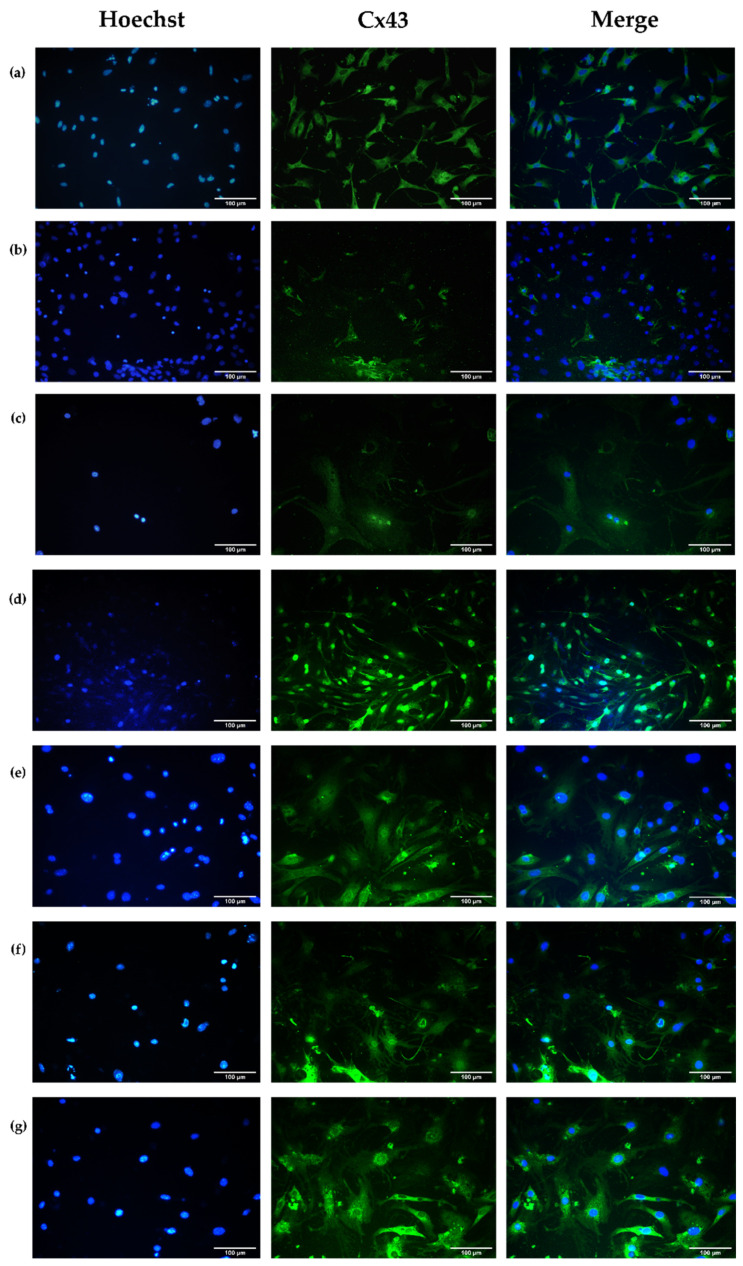
Representative micrographs of primary fetal ventricular cardiomyocytes expressing the Cx43 protein under conditions of (**a**) normoxia, (**b**) ischemia, (**c**) ischemia/reperfusion (I/R), (**d**) ischemia/intervention with B1 hydrogel (I/B1), (**e**) ischemia/intervention with the B2 hydrogel (I/B2), (**f**) ischemia/intervention with encapsulated cells in B1 hydrogel (I/B1/C), (**g**) ischemia/intervention with encapsulated cells in B2 hydrogels (I/B2/C). B1: Ch 2.5% *w*/*v*/β-GP 10.5% *w*/*v*/HcB 6.5% *w*/*v*. B2: Ch 2.5% *w*/*v*/β-GP 10.5% *w*/*v*/HcP 6.5% *w*/*v*. Evaluation at 72 h. Scale bar: 100 µm.

Figure 12 shows the expression levels of the Cx43 of primary ventricular cardiomyocytes from mice under different intervention scenarios. As a general condition, Cx43 is expressed throughout a large part of the cell body and is not limited to specific intracellular sites.

After the induced ischemia process (Figure 12b), the Cx43 expression level decreased compared to cultures in normoxia (Figure 12a) because of the modulation of protein phosphorylation and their localization in the plasma membrane or sarcolemma due to the decrease in ATP [45], and the increase in acidosis and intracellular Ca^2+^ [41], among other cellular alterations caused by hypoxia. Cx43 has a half-life of approximately 1.5–2 h [46], and the phosphorylation of its C-terminus occurs post-translationally [47], meaning that, at an experimental time of 3 h, at low concentrations of oxygen and glucose, a high Cx43 percentage should be obtained following the phosphorylation of its serine residues. It was demonstrated that hypoxia times longer than 15 min decrease the synthesis of the Cx43 protein, and its post-translational phosphorylation can be increased or decreased in some serine residues, which affects the formation of gap junctions and their localizations in the sarcolemma, allowing the protein to either be removed by ubiquitination or internalized in and localized to areas that do not contribute to intercellular communication [48].

Following ischemia, the cells experienced different interventions (Figure 12c–g) to determine the Cx43 expression restoration outcomes. Cells that were exposed to I/R processes (Figure 12c) showed an increase in protein expression compared to the post-ischemic event (Figure 12b); however, a decrease in cell density was observed, which could be due to the rapid restoration of normoxic conditions after the ischemic period. It has been reported that the restoration to normal conditions of cell cultures increases the ROS and further mitochondrial damage [49].

The four types of post-ischemic hydrogel interventions (Figure 12d–g) increased the Cx43 expression concerning cell control (Figure 12a) and I/R processes (Figure 12c). These results can be associated with the fact that chitosan, when in contact with cultured cells, favors the production of collagen type I [29]. Collagen is part of the ECM, and the interaction between cells with ECM regulates the expression of connexins and their intracellular distribution and functionality by forming hemichannels or gap junctions [50].

The results in Figure 12d,e are comparable with the results in Figure 12f,g, where the intervention includes encapsulated healthy cells where an increase in the expression of the Cx43 protein is also reflected and can be indicative of the integration of cells that are part of the treatment with those in the cell culture.

##### Alpha-Actinin

α-Actinin is an actin-binding protein essential to the contraction process of cardiac muscle cells. This protein is expressed in non-muscle cells and striated and smooth muscle cells [51]. In skeletal muscles, such as cardiac muscle, α-actinin cross-links actin filaments to adjacent sarcomeres, playing a role in loading and force transmission in cardiomyocytes. α-Actinin and other proteins are part of a complex network called the Z line that stabilizes the actin filaments to interact with other elements that allow for muscle contractions. A-Actinin also binds to molecules, such as phosphatidylinositol-bisphosphate (PIP2), integrins, PI3K (signaling enzyme), and vinculin, thereby regulating intercellular adherent junctions [52].

**Figure 13:**
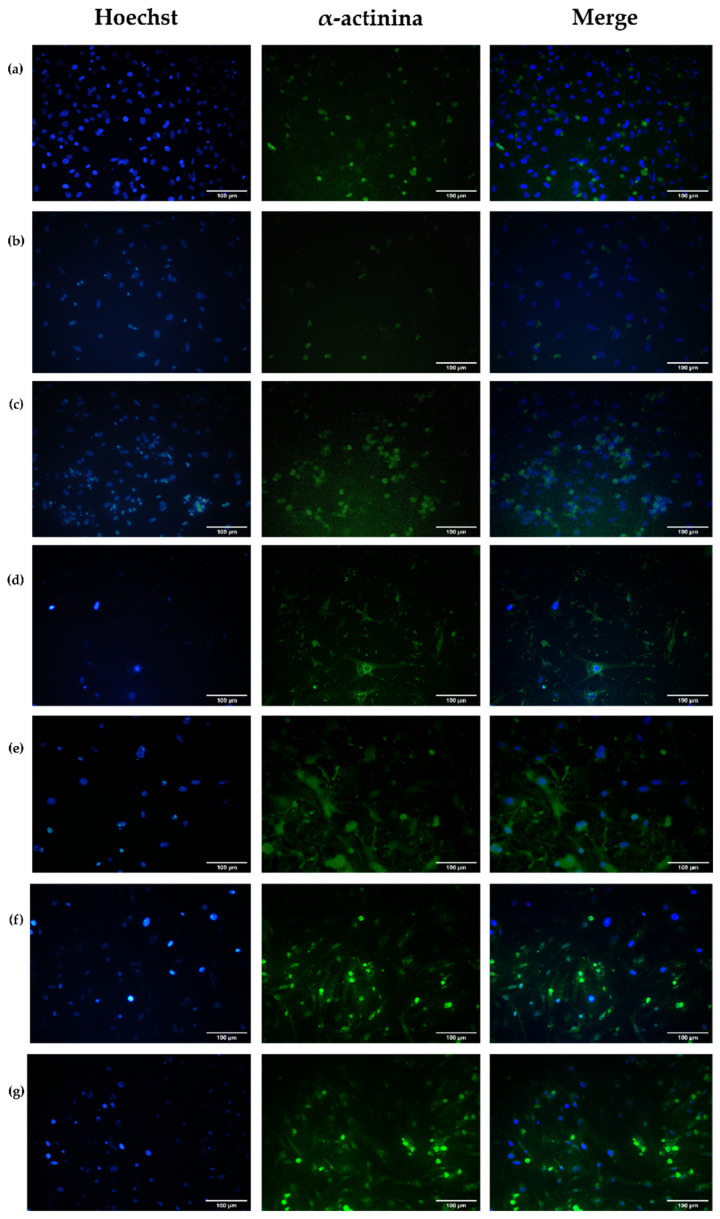
Representative micrographs of primary fetal ventricular cardiomyocytes expressing the α-actinin protein under conditions of (**a**) normoxia; (**b**) ischemia; (**c**) ischemia/reperfusion (I/R); (**d**) ischemia/intervention with B1 hydrogel (I/B1); (**e**) ischemia/intervention with B2 hydrogel (I/B2); (**f**) ischemia/intervention with B1 hydrogel, which contains cells in encapsulated (I/B1/C); (**g**) ischemia/intervention with the B2 hydrogel, which contained encapsulated cells (I/B2/C). B1: Ch 2.5% *w*/*v*/β-GP 10.5% *w*/*v*/HcB 6.5% *w*/*v*. B2: Ch 2.5% *w*/*v*/β-GP 10.5% *w*/*v*/HcP 6.5% *w*/*v*. Evaluation at 72 h. Scale bar: 100 µm.

Figure 13 shows the α-actinin expression in primary ventricular cardiomyocytes under different interventions. Following the ischemia process (Figure 13b), the expression of α-actinin decreases, regarding to cells under normoxic conditions (Figure 13a). This α-actinin protein expression decrease is related to both myofilament regulatory proteins and structural and cytoskeletal proteins vulnerable to cleavage or loss under mild ischemia/reperfusion conditions. In these cases, microtubule rupture and the loss of α-actinin and other proteins, such as desmin and spectrin, occur [53]. These changes in the myofilament structure associated with ischemia/reperfusion are correlated with myofilaments’ reduced capacity to develop force against and sensitivity to Ca^2+^, causing cell density loss.

Following ischemia, the cells experienced different treatments (Figure 13c–g) to determine the α-actinin expression recovery rate. The reperfused cells (Figure 13c) showed increased protein expression levels compared to the post-ischemic cells (Figure 13b), without a decrease in the cell density. The post-ischemic interventions with the hydrogels (Figure 13d–g) increased the expression of the α-actinin protein concerning cellular control (Figure 13a) and I/R (Figure 13c).

For the cells intervened with encapsulated cells (Figure 13f–g), an increase in the α-actinin expression protein is presented for the cell density increases, and more intercellular connections are established. Therefore, the sarcomeres are increased and strengthened.

#### 3.4.4. Intracellular Calcium Evaluation

The electrophysiological coupling of the cardiac cell implies a constant movement of ions through the plasmatic membrane that activates the calcium channels and allows the entrance of this ion into the cell so that the cell contraction process can occur. However, when cells are in conditions of low oxygen and glucose concentrations, cell metabolism is affected because, in the absence of oxygen, the cell shifts from obtaining energy aerobically to anaerobically, which leads to an increase in intracellular Na^+^. Through the Na^+^/Ca^2+^ exchanger (the transmembrane protein channel that, under normal conditions, allows calcium exit), Na^+^ leaves the cell again, allowing for the entry of Ca^2+^. In this way, it significantly increases the levels of intracellular Ca^2+^, activating the ryanodine receptor, which releases more calcium from the sarcoplasmic reticulum into the cytoplasmic space. In a healthy functional cell, the influx of Ca^2+^ drives the cell into cycles of contraction and relaxation; however, Ca^2+^ overload causes irreversible damage to the cell, such as contractile dysfunction and apoptosis. In the mitochondria, there may also be an uncontrolled accumulation of Ca^2+^ due to the ischemic event, where it acts on different enzymes and proteins necessary for the tricarboxylic acid cycle and ATP production [54].

Considering the importance of Ca^2+^ in cardiac cell physiology, calcium flux was determined in cultures of healthy primary cardiomyocytes and under hypoxic conditions using the Indo-1 AM radiometric probe. This probe is cleaved by intracellular esterase when it enters the cell, leaving the Indo-1 group free to be bound to the intracellular calcium available in the cytoplasmic space [54,55].

**Figure 14:**
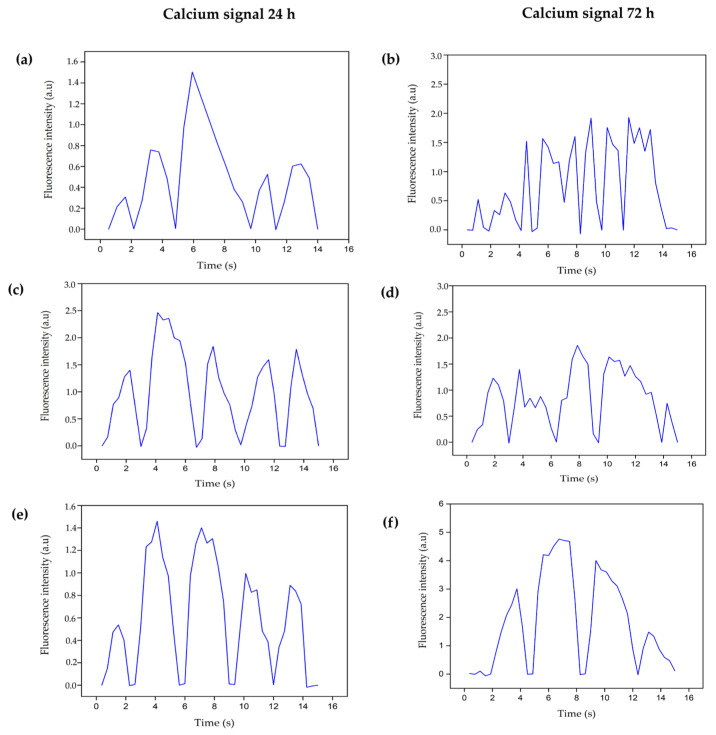
Calcium fluorescence intensities of fetal ventricular cardiomyocytes were recorded at 24 and 72 h post-seeding. (**a**) Healthy cells at 24 h, (**b**) healthy cells at 72 h, (**c**) healthy cells at 24 h intervened with B1 hydrogel, (**d**) healthy cells at 72 h intervened with B1 hydrogel, (**e**) healthy cells at 24 h intervened with B2 hydrogel, (**f**) healthy cells at 72 h intervened with B2 hydrogel. B1: Ch 2.5% *w*/*v*/β-GP 10.5% *w*/*v*/HcB 6.5% *w*/*v*. B2: Ch 2.5% *w*/*v*/β-GP 10.5% *w*/*v*/HcB 6.5% *w*/*v*.

Figure 14 shows the Ca^2+^ signals emitted by healthy cells and healthy cells with the B1 and B2 hydrogels. Figure 14a, shows the basal Ca^2+^ fluorescence intensity at 24 h, which increases and decreases throughout 15 s time period. The observed complexes indicate the flow of extracellular Ca^2+^ entering the cell and the exit of intracellular Ca^2+^ from the sarcoplasmic reticulum to the cell cytoplasm. All calcium molecules found in the cytoplasm bind to the fluorescent molecule. The observed decrease in the fluorescence intensity occurs due to the closure of calcium ion channels in the plasma membrane or ryanodine channels in the sarcoplasmic reticulum that prevent Ca^2+^ from exiting into the cytoplasmic space. Therefore, no calcium molecules are bound to the probe and the fluorescence intensity is decreased.

In Figure 14d, the complexes increase in number and intensity because, at 72 h, the cell density is higher, as evidenced in the upper left micrograph.

Healthy cells were intervened with B1 and B2 hydrogels to determine the basal Ca^2+^ influx in contact with the hydrogels. Figure 14b shows how the cell Ca^2+^ signal intensity interacting with B1 increases from a maximum of 1.5 to 2.5 at 24 h; in the same way, at 72 h (Figure 14e), some complexes with low intensities around 0.5 increase to values close to 1.5.

The same increase in the fluorescence intensity effect was observed in cells that were in contact with the B2 hydrogel (Figure 14c–f), indicating that both types of biomaterials favored the entry of Ca^2+^ from the extracellular medium and the exit of Ca^2+^ from the sarcoplasmic reticulum to the cytoplasmic space, where it could be bound to troponin C filaments and promote cell contraction, since Ca^2+^ exposed the actin active site that bound to myosin [55].

**Figure 15:**
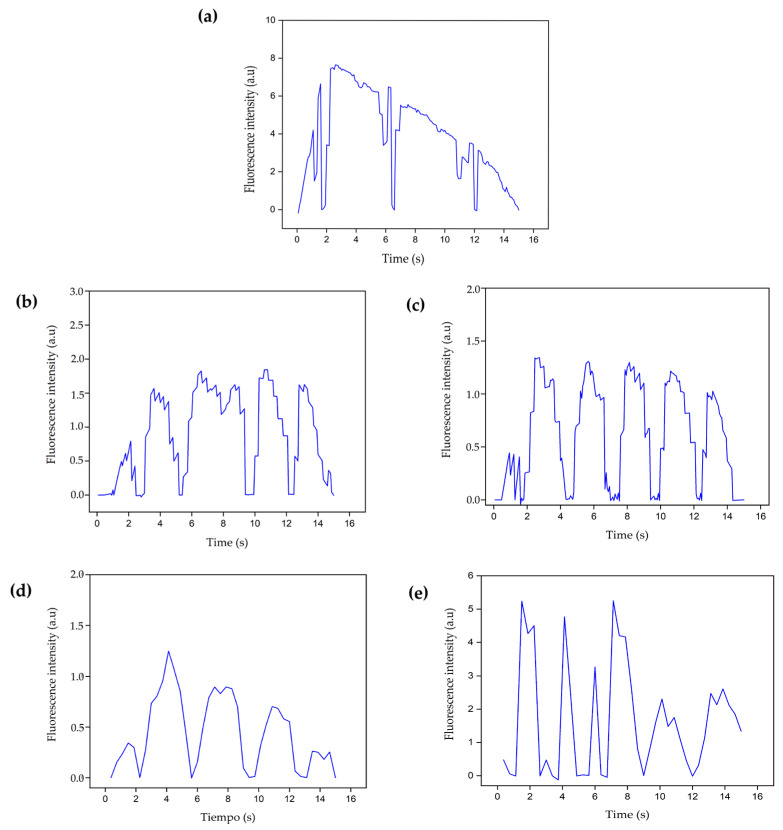
Calcium fluorescence intensities of fetal ventricular cardiomyocytes were recorded 24 h post-seeding. (**a**) Ischemic cells (control), (**b**) ischemic cells intervened with B1 hydrogel, (**c**) ischemic cells intervened with encapsulated cells in B1 hydrogel, (**d**) ischemic cells intervened with B2 hydrogel, (**e**) ischemic cells intervened with encapsulated cells in B2 hydrogel. B1: Ch 2.5% *w*/*v*/β-GP 10.5% *w*/*v*/HcB 6.5% *w*/*v*. B2: Ch 2.5% *w*/*v*/β-GP 10.5% *w*/*v*/HcB 6.5% *w*/*v*.

**Figure 16:**
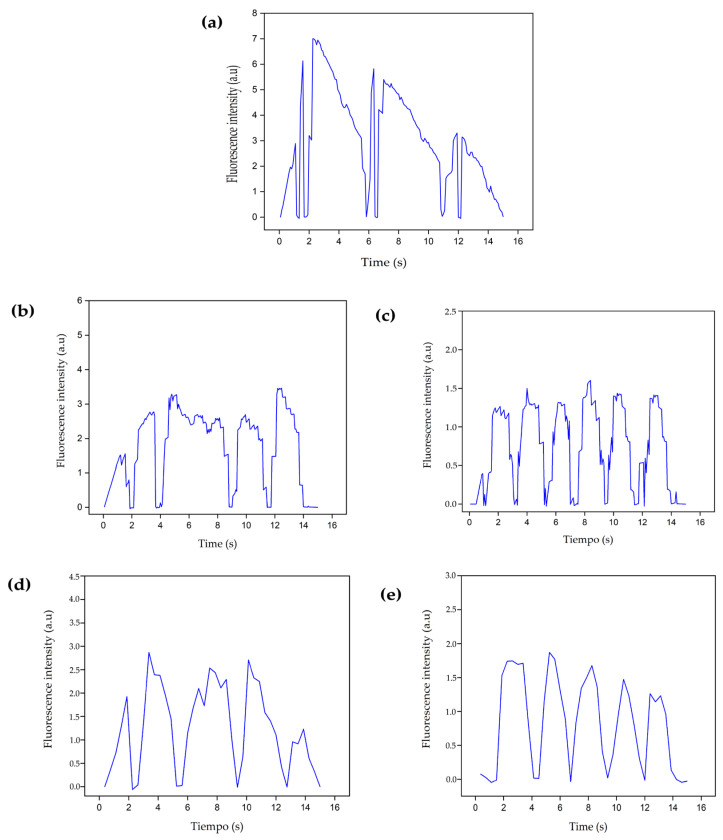
Calcium fluorescence intensities of fetal ventricular cardiomyocytes were recorded 72 h post-seeding. (**a**) Ischemia control, (**b**) Ischemic cells intervened with B1 hydrogel, (**c**) Ischemic cells intervened with encapsulated cells in B1 hydrogel, (**d**) Ischemic cells intervened with the B2 hydrogel, (**e**) Ischemic cells intervened with encapsulated cells in B2 hydrogel. B1: Ch 2.5% *w*/*v*/β-GP 10.5% *w*/*v*/HcB 6.5% *w*/*v*. B2: Ch 2.5% *w*/*v*/β-GP 10.5% *w*/*v*/HcB 6.5% *w*/*v*.

Figure 15 and Figure 16 show the calcium influx levels at 24 and 72 h respectively, for control and ischemic cells intervened with B1 and B2 hydrogels or with cells encapsulated in the hydrogels.

The images in Figure 15 and Figure 16 correspond to the ischemic cells evaluated at 24 and 72 h, respectively. The graphs show how the calcium influx increases to maximum values close to 7.0, that is, a maximum increase in fluorescence over 450%, exceeding the values observed in healthy cells (Figure 14a–d). This effect occurs due to the alteration in the ion channels of the plasma membrane and the continuous entry of extracellular calcium, which in turn stimulates the release of calcium from the sarcoplasmic reticulum. This imbalance in the intracellular calcium concentrations does not favor cell dynamics, and finally, apoptosis-type cell death processes are triggered [18]. In addition, the graphs show that the complexes are more durable over time, so cell repolarization occurs more slowly, allowing more calcium to enter the cell, favoring the overload of this molecule intracellularly. On the other hand, there is no increase in the number of complexes, as it occurs in cells intervened with hydrogels B1 and B2 (Figure 14), where complete cycles of the opening and closing of ion channels are favored; however, in ischemic cells, the ion channels remain open longer due to the ischemic process that alters cell metabolism.

At 24 h, cells intervened with B1 and B2 hydrogels decreased in fluorescence intensity (Figure 15b–d) compared to the ischemic cells (Figure 15a), reaching values close to those found in the control of healthy cells (Figure 14a). At 72 h (Figure 16b–d), the fluorescence intensity also decreased compared to the ischemia control; however, it was higher at 24 h and concerning healthy cells (Figure 15b). As initially observed in the healthy cells, the longer the culture time, the higher the fluorescence intensity and the more complexes are formed. In the same way, the interaction time with the hydrogel is essential for cell recovery after an ischemic event. In both periods, maximum calcium values were achieved, close to the values obtained for healthy cells, that is, the ischemic cells treated with the hydrogels had the possibility of recovering the basal calcium influx, which also increased the probability of recovering the calcium-dependent cell contraction affected by the ischemic event.

The effect of the encapsulated cells on the recovery of the basal calcium influx in the ischemic cells was similar to the intervention effect that only included the hydrogel, as shown in Figure 15c–e and Figure 16c–e. However, there was an increase in the number of complexes, that is to say, a higher number of calcium molecules joined the probe simultaneously, more cellular excitation occurred, and the contraction frequency increased due to the increase in the cell density due to the intervention. These results indicate the time-dependent electrophysiological coupling activity between the encapsulated and culture cells.

## 4. Conclusions

Two types of hydrogels of natural origins were developed. The hydrogels were produced from hydrolyzed collagen, chitosan and beta-glycerol phosphate, which responded to changes in temperature, being liquid at room temperature and gelling at temperatures equal to or higher than 37 °C. These hydrogels had an internal three-dimensional structure of interconnected pores, allowing for cell encapsulation. The hydrogels progressively degraded in a physiological environment over time. In addition, the hydrogels were not cytotoxic in the interaction with fetal ventricular cardiomyocyte primary cultures, did not lyse red blood cells, and could restore contractile activity in ischemic cardiomyocytes when used as an intervention method alone or in combination with cardiac progenitor cells in their internal structure.

Considering the results for both hydrogels, sample B1 exhibits better bio- and heme-compatible properties. In the same way, when biofunctionalizing this hydrogel with cardiac progenitor cells, the restoration of the contractility of ischemic cardiomyocytes is similar to cell control. Therefore, the B1 hydrogel will continue to be used in subsequent studies where pathological models will involve three-dimensional cardiac cultures and an ex vivo model of an isolated and perfused heart.

## Figures and Tables

**Table 1:** Hemolytic index and degree accepted by the ISO 10993-4 standard for the direct contact hemolysis test.

Hemolytic Index	Hemolytic Degree
0–2	Non-hemolytic
2–5	Slightly hemolytic
>5	Hemolytic

## Data Availability

Data are contained within the article.
